# Genome-wide analysis of terpene synthase gene family to explore candidate genes related to disease resistance in *Prunus persica*

**DOI:** 10.3389/fpls.2022.1032838

**Published:** 2022-10-31

**Authors:** Xiongwei Li, Yang Hu, Mingshen Su, Minghao Zhang, Jihong Du, Huijuan Zhou, Xianan Zhang, Zhengwen Ye

**Affiliations:** ^1^ Forest and Fruit Tree Institute, Shanghai Academy of Agricultural Sciences, Shanghai, China; ^2^ Shanghai Runzhuang Agricultural Science and Technology Co., Ltd, Shanghai, China

**Keywords:** peach (*Prunus persica* L.), terpene synthase genes, gummosis disease, terpenoids, linalool

## Abstract

In plants, a family of terpene synthases (TPSs) is responsible for the biosynthesis of terpenes and contributes to species-specific diversity of volatile organic compounds, which play essential roles in fitness of plants. However, little is known about the *TPS* gene family in peach and/or nectarine (*Prunus persica* L.). In this study, we identified 40 *PpTPS* genes in peach genome v2.0. Although these *PpTPSs* could be clustered into five classes, they distribute in several gene clusters of three chromosomes, share conserved exon-intron organizations, and code similar protein motifs. Thirty-five *PpTPSs*, especially *PpTPS2*, *PpTPS23*, *PpTPS17*, *PpTPS18*, and *PpTPS19*, altered their transcript levels after inoculation with *Botryosphaeria dothidea*, a cause of peach gummosis, compared to the mock treatments, which might further affect the contents of 133 terpenoids at 48 hours and/or 84 hours post inoculations in the current-year shoots of ‘Huyou018’, a highly susceptible nectarine cultivar. Moreover, about fifteen *PpTPSs*, such as *PpTPS1*, *PpTPS2*, *PpTPS3*, and *PpTPS5*, showed distinct expression patterns during fruit development and ripening in two peach cultivars, yellow-fleshed ‘Jinchun’ and white-fleshed ‘Hikawa Hakuho’. Among them, the transcription level of chloroplast-localized *PpTPS3* was obviously related to the content of linalool in fruit pulps. In addition, elevated concentrations (0.1 g/L to 1.0 g/L) of linalool showed antifungal activities in PDA medium. These results improve our understanding of peach *PpTPS* genes and their potential roles in defense responses against pathogens.

## Introduction

Volatile organic compounds (VOCs) are essential for the interaction of plants with pollinators, seed-spreaders, herbivores, and microorganisms (Dudareva et al., 2013; Bouwmeester et al., 2019). Among the four classes of plant VOCs (terpenoids, phenylpropanoids/benzenoids, fatty acid derivatives, and amino acid derivatives), terpenoids constitute the largest and most diverse class of secondary metabolites with many volatile constituents (Dudareva et al., 2006; Dudareva et al., 2013). Terpenoids are derived from two common C_5_-isoprene building units, isopentenyl diphosphate (IPP) and its allylic isomer, dimethylallyl diphosphate (DMAPP) (McGarvey and Croteau, 1995). Both IPP and DMAPP are substrates for short-chain prenyltransferases, which produce prenyl diphosphate precursors, farnesyl diphosphate, geranyl diphosphate, and geranylgeranyl diphosphate (Vranova et al., 2013). A family of terpene synthases/cyclases (TPSs) is responsible for the synthesis of volatile terpenoids from prenyl diphosphate precursors in cytosol and/or plastids (Chen et al., 2011). Although two compartmentally separated pathways, mevalonic acid (MVA) and methylerythritol phosphate (MEP), are responsible for the formation of IPP and DMAPP in plants, cross-flows between MVA and MEP pathways have been identified (Vranova et al., 2013). Therefore, the divergent function and subcellular localization of TPSs determine the diversity of volatile terpenoids. In general, through specific activities of TPSs, the MVA pathway generates volatile sesquiterpenes (C_15_) in cytosol and peroxisome, whereas the MEP pathway generates volatile hemiterpenes (C_5_), monoterpenes (C_10_), and diterpenes (C_20_) in plastids (McGarvey and Croteau, 1995; Sapir-Mir et al., 2008; Vranova et al., 2013).

*TPS* genes have been widely found in bacteria, fungi, and plants, but their proteins show high structural similarity and share a common evolutionary origin. Notably, the size of the *TPS* gene family shows divergence in different lineages (Chen et al., 2011). For example, only a single functional *TPS* gene, coding a copalyl synthase/kaurene synthase (CPS/KS), was found in the bryophyte *Physcomitrella patens*, but over 100 *TPS* gene models were found in *Vitis vinifera* (Hayashi et al., 2006; Martin et al., 2010). Usually, a typical/complete TPS protein consists of two independent domains at the N-terminal and C-terminal. The N-terminal domain of the protein has features of the active site of a CPS, whereas the KS activity is located in the C-terminal of the protein (Hayashi et al., 2006). In most cases, functional *TPS* genes lose activity in either the CPS- or KS-type domains in both gymnosperms and angiosperms, that is, these TPSs only act as monofunctional TPSs, whereas the unique TPS in *P. patens* is a bifunctional enzyme (Keeling et al., 2010). This gene family is divided into seven subfamilies (TPS-a through TPS-g) based on sequence relatedness, functional assessment, and gene architecture (Aubourg et al., 2002; Alquezar et al., 2017; Chen et al., 2017; Yu et al., 2020; Zhou et al., 2020). TPSs from related plant species tend to cluster more than enzymes with similar functions, thus challenging the substrate/product predictions based on sequence similarities (Bohlmann et al., 1998; Aubourg et al., 2002). These results suggest that the expansion of *TPS* gene family can be species- and/or lineage-specific. As a result, the majority of *TPS* genes are generated through tandem duplications rather than whole-genome duplications or segmental duplications (Yu et al., 2020; Zhou et al., 2020). Moreover, the roles of some TPS proteins can be variable and associated with the availability of substrates and the microenvironment of distinct compartments (Aharoni et al., 2004). For example, two sesquiterpene synthases, TPS21 and TPS11, account for the biosynthesis of nearly all 20 sesquiterpenes found in the floral volatile blend in *Arabidopsis* (Tholl et al., 2005).

Since terpenoid-derived VOCs are pivotal to the survival and fitness of plants, the importance and potential roles of TPSs in the biosynthesis of VOCs and highly complicated interactions/conversations (ecological correlations) between plants and other living beings, including humans, animals, plants, and microbes have attracted attention (Dudareva et al., 2013; Song et al., 2018; Huang et al., 2019). Conversely, TPSs predominantly affect the overall flower and fruit aroma, which determines the multiplication capacity and economic value of plants. For example, *Cstps1*, a sesquiterpene synthase encoding-gene, is involved in the production of valencene, the key aroma compound in *Citrus* fruits (Sharon-Asa et al., 2003). The evolution of *TPS20*-related terpene synthases influences chemical diversity in the glandular trichomes of the wild tomato relative *Solanum habrochaites* (Gonzales-Vigil et al., 2012). The sequence variation of *Fragaria ananassa Nerolidol Synthase1* (*FaNES1*) gene in cultivated strawberry might be the genetic basis of fruit flavor selections, which contributes to the higher levels of linalool and nerolidol than wild *F. vesca* (Aharoni et al., 2004). Meanwhile, TPSs provide basal products for the biosynthesis of toxic metabolites against undesirable invaders. For example, rice *OsTPS24* encodes a jasmonate-responsive monoterpene synthase that produces an antibacterial *ɤ*-terpinene against *Xanthomonas oryzae* pv. *oryzae* (*Xoo*), but it does not show significant antifungal activity against the blast fungus *Magnaporthe oryzae* (Yoshitomi et al., 2016). However, rice terpene synthase gene *OsTPS19* functions as an (*S*)-limonene synthase in plants and its overexpression leads to enhanced resistance to *Magnaporthe oryzae* (Chen et al., 2018). In addition, *OsTPS46*, a rice terpene synthase, confers natural resistance to bird cherry-oat aphid (Sun et al., 2017). In soybean, *GmAFS* encodes an (*E*, *E*)-α-farnesene synthase and has defensive roles in both below-ground and above-ground organs of soybean against nematodes and insects, respectively (Lin et al., 2017).

Peach (*Prunus persica* L.), which has flavorful aroma, is a popular and economically important fruit crop worldwide. However, because of the absence of efficient genetic resistance, most peach cultivars suffer from the destructive gummosis disease in high temperature and humidity areas, such as Japan, south of China and USA. Fungal peach gummosis is mainly caused by *Botryosphaericeae* pathogens (*Lasiodiplodia theobromae*, *Botryosphaeria dothidea*, *Diplodia seriata*, *Neofusicoccum parvum*) (Wang et al., 2011; Ye et al., 2020). Although there were several studies elucidated the roles of *TPSs* in flavour formation of peach fruit, little is known about their function in defense responses (Liu et al., 2017; Wei et al., 2021; Wei et al., 2022). In this study, the genome-wide identification and preliminary bioinformatics analyses of *PpTPS* genes were performed. An ‘omics’ approach combining non-targeted metabolomics based on ultra-high performance liquid chromatography-mass spectroscopy (UHPLC-MS), gas chromatography-mass spectroscopy (GC-MS) and transcriptome was performed, the aim is to investigate the potential roles of *PpTPS* genes and terpenes in defense responses of peach plants to pathogens.

## Materials and methods

### Identification of *PpTPS* gene family in the genome of *Prunus persica*

Genome sequences of *Prunus persica* were downloaded from Genome Database for Rosaceae (https://www.rosaceae.org/species/prunus_persica/genome_v2.0.a1). The hidden Markov model profile of the metal binding domain (PF01397), C-terminal metal binding domain (PF03936), and N-terminal domain (PF19086) was downloaded from the Pfam database (http://pfam.xfam.org). Redundant sequences were deleted using Blastp, and only the longest sequences among alternative splicing variants were retained.

### Phylogenetic tree construction and bioinformatics analyses

The amino acid sequences of TPSs with defined functions in rice (*Oryza sativa*), apple (*Malus domestica*), grapevine (*Vitis vinifera*), strawberry (*Fragaria ananassa* and *F. vesca*), kiwifruit (*Actinidia chinensis* and *A. arguta*), pineapple (*Ananas comosus*), poplar (*Populus trichocarpa*), cotton (*Gossypium hirsutum*), sweet orange (*Citrus sinensis*), and *Arabidopsis* were downloaded from the National Center for Biotechnology Information (NCBI) according to the accession number of each gene (Aharoni et al., 2004; Tholl et al., 2005; Martin et al., 2010; Nieuwenhuizen et al., 2013; Nieuwenhuizen et al., 2015; Yoshitomi et al., 2016; Alquezar et al., 2017; Chen et al., 2017; Sun et al., 2017; Chen et al., 2018; Huang et al., 2018). These sequences were used to generate a phylogenetic tree based on Multiple Sequence Comparison by Log-Expectation (MUSCLE) alignment, then phylogenetic and molecular evolutionary analyses were conducted using MEGA version 11 (Tamura et al., 2021). Neighbor-joining analysis with pairwise deletion was performed using the Jones-Taylor-Thornton model. Bootstrap analysis was performed with 1000 replicates to assess the level of statistical support for each tree node. The putative protein domains of PpTPSs were identified using the MEME Suite (http://meme-suite.org/meme), while the gene structures of *PpTPSs* were analyzed using the Gene Structure Display Server (http://gsds.gao-lab.org). The upstream 1.5 kb region of the translation start site of the *PpTPS* genes was used for putative promoter *cis*-acting element analysis in PlantCARE (http://bioinformatics.psb.ugent.be/webtools/plantcare/html). The motifs putatively involved in different types were summarized. For synteny analysis, synteny blocks between peach and *Citrus sinensis* and *Arabidopsis thaliana* genomes were obtained from the Plant Genome Duplication Database (http://chibba.agtec.uga.edu/duplication/index/downloads).

### Quantification of linalool in fruit pulp of cv. ‘Jinchun’ and ‘Hikawa Hakuho’ by GC-MS

Two early-maturity cultivars with different fruit flesh colors, ‘Jinchun’ (yellow-fleshed) and ‘Hikawa Hakuho’ (white-fleshed), were selected to investigate the accumulation of linalool and transcription change of *PpPTS* genes during developmental stages. Three trees for each cultivar were grown under consistent and standard agronomic practices for fertilization, irrigation, fruit thinning, pruning, and pest and disease control in the Zhuanghang experimental field of Shanghai Academy of Agricultural Sciences (SAAS), Shanghai, China (121.45°E, 30.92°N). Fruits were collected at five sampling times (day 35 (S1), 50 (S2), 65 (S3), 80 (S4), 90 (S5), 95(S6)) after full blossom. For each sampling stage, 20 fruits were selected and taken to the laboratory immediately. The mesocarp of each fruit were cut into small pieces and frozen instantly in liquid nitrogen and stored at -80°C for linalool quantification and RNA sequencing. Linalool was quantified using a modified method based on the description of Liu and Wei et al. (Liu et al., 2017; Wei et al., 2021).

### Pathogen inoculation assays and measurement of terpenoids in shoots *via* UHPLC-MS

Three pathogens isolations, designated as *Botryosphaeria dothidea* SHTLJ001 (OP340986), *Botrytis cinerea* SHTLK001 (OP340985), and *Monilinia fruticola* SHTHF001 (OP340987) were collected from the peach experimental trial fields of Shanghai Academy of Agricultural Sciences, which were purified through multiple subculture of hyphae and identified by sequencing of their ITSs with common primers *ITS1* and *ITS4.* The current-year shoots of the susceptible cultivar ‘Huyou018’ were inoculated with *B. dothidea* SHTLJ001 using the method described in Gao et al. (Gao et al., 2016). In brief, the surface-sterilized peach shoots were cut into 15 cm-long segments and then wounded with a sterilized needle. A single mycelial plug (5 mm in diameter) of *B. dothidea* SHTLJ001 was placed onto the wound point. Shoot segments inoculated with sterile PDA medium without *B. dothidea* SHTLJ001 were treated as controls. The inoculated and control shoots were placed in square plastic boxes laying multiple layers of wet filter papers. The boxes were covered with clear plastic wrap to keep 90% relative humidity and then placed in a light incubator at 28/25°C, with a photoperiod of 12/12 h light (5000 lux)/dark.

To measure the relative contents of terpenoids in peach shoots post *B. dothidea* inoculations, the tissue measuring 0.5 cm in diameter was cut from the lesion area and frozen at -80°C at 0 h, 48 h, and 84 h after inoculation. The extraction and relative quantification of terpenoids was performed according to the method described in Sawada et al. (Sawada et al., 2009). Briefly, 50 mg of freeze-dried and ground sample was transferred to a 2 mL tube, and 700 μL of extract solution (methanol/water=3:1, precooled at -40°C, containing internal standard) was added. After being vortexed for 30 s, the samples were homogenized at 35 Hz for 4 min and sonicated for 5 min in an ice-water bath. Homogenization and sonication were performed three times. After being extracted overnight at 4°C on a shaker, the samples were centrifuged at 12,000 rpm for 15 min at 4°C. The supernatant was carefully filtered through a 0.22 μm microporous membrane, and then the resulting supernatants were diluted 20 times with methanol/water mixture (v:v=3:1, containing an internal standard), vortexed for 30 s, and transferred to 2 mL glass vials. Next, 40 μL of each sample was pooled as quality control samples. The samples were used to perform UHPLC-MS analyses with previous methods (Sawada et al., 2009).

### RNA-seq of shoot tissue after inoculation with pathogens and fruit pulp at different developmental and ripening stages

Total RNA extraction and first-strand complementary DNA (cDNA) synthesis for both shoots and fruit pulp were carried out using the method described in Li et al. (2015). The construction of RNA sequencing libraries and the quantification of gene expression level were generated according to previous study (Pertea et al., 2015). The FPKM of each gene was calculated based on the length of the gene and the read count mapped to that gene. The change of gene transcription level in each stage was compared with the first sampling stage.

### Subcellular location of PpTPSs in the leaves of *Nicotiana benthamiana*

The CDS of PpTPS1, PpTPS3, and PpTPS4 were cloned from the cDNA library of ‘Huyou018’ with gene-specific primers (**Supplementary Table 1**). Then, the comfirmed CDS sequences were subcloned into pCambia1302-GFP vector. The fusion proteins were transiently expressed in the fully expanded young leaves of six-week-old *Nicotiana benthamiana via Agrobacterium*-midiated infiltration (Wydro et al., 2006). Images were obtained with a LEICA laser scaning confocal microscope at 3 days post infiltrations.

### Antifungal activity of linalool in PDA medium

To test the antifungal activities of linalool, the growth speed assays of three fungi species were performed on PDA solid medium containing different concentrations of linalool. The pure linalool (ANPEL Laboratory Technologies, Shanghai) were dissolved in the 1 mL ethanol (Sinopharm Chemical Reagent Co., Ltd, Shanghai) to prepare a mixture liquid. Then, different volumes of mixture liquid were added into 200 mL sterilized PDA medium (prior to solidification). The equal amount of fresh hyphal mass were inoculated at the center of new PDA plates with a needle and the diameter of colony were measured at 3 days post inoculations.

### Statistical analysis

The experiment was arranged in a completely randomized design with three replications, and collected data were statistically analyzed using multivariate analysis methods in SAS computer software (SAS Version 9.2, Institute). Analysis of variance (ANOVA) was used to determine the overall statistical significance of the data at the significance level of *P* <0.05, and the data were represented as average ± STDEV (*n* = 3). The heatmaps were visualized using TBtools software (Chen et al., 2020).

## Results

### Identification and classification of *PpTPS* genes in *Prunus persica*

Based on the genome sequences of peach (Lovell v2.0.a1), a total of 45 candidate *TPS* genes were preliminarily identified, and their deduced proteins contain at least one of the three core Pfam domains (PF01397, PF03936, and PF19086) that are essential to the catalytic activities of TPS. Five of them (Prupe.4G193900, Prupe.4G194000, Prupe.4G194700, Prupe.4G238800, and Prupe.4G265600) were too short (the length of amino acid sequences is shorter than 250) to code a single functional domain in length; therefore, they were discarded. There were three *PpTPS* genes (Prupe.4G030400, Prupe.4G029900, and Prupe.4G030300) have been named as *PpTPS1*, *PpTPS2* and *PpTPS3* in previous studies (Liu et al., 2017; Wei et al., 2021). According to the chromosome localization of the genes, the remaining 37 peach *TPS* genes were named as *Prunus persica Terpene Synthases* 4 to 40 (*PpTPS4*-*PpTPS40*) (**Table 1**). These *PpTPS* genes are only distributed in three chromosomes of peach (chromosome 3, 4, and 8). Among them, chromosome 4 contains 34 *PpTPSs*, which intensively occur in several gene clusters, whereas chromosome 8 only has 1 *PpTPS* (*PpTPS40*) (**Table 1**). The phylogenetic analysis grouped these 40 *PpTPSs* into 5 clades corresponding to the TPS classes a (22), b (8), c (1), e/f (7), and g (2) (**Figure 1A**). The synteny analysis of *TPS* genes among *P. persica*, *A. thaliana*, and *C. sinensis* suggested that only few *PpTPS* genes showed synteny relations and the Ka/Ks ratio of these synteny genes are far less than 1.0 (**Figure 1B**, **Supplementary Table 2**), which means that most of these *PpTPS* genes were generated after lineage separation and those synteny genes have undergone purifying selections. Detailed information concerning each putative *PpTPS* gene is presented in **Table 1**.

**Table 1:** Information of terpene synthase genes in peach (*Prunus persica*).

Gene name	Accession no.	Gene size	Location	Deduced polypeptide					
				No. of aa	Subgroup	DDXXD	NSE/DTE	DXDD	Subcellular localization^#^
PpTPS1*	Prupe.4G030400	2557	Pp04:1419829.1422386	579	TPS-g	DDIFD	DDLGTAEDE	—–	**Chloroplast**
PpTPS2*	Prupe.4G029900	2709	Pp04:1390452.1393161	575	TPS-b	DDVYD	NDLGTSAAE	—–	**Cytoplasm**
PpTPS3*	Prupe.4G030300	2602	Pp04:1413599.1416201	535	TPS-g	DDIFD	DDFGSAKDE	—–	**Cytoplasm**
**PpTPS4**	Prupe.3G222200	4107	Pp03:22262877.22266984	629	TPS-b	DDMYD	DDLGTSKAE	—–	Chloroplast
**PpTPS5**	Prupe.3G222300	3364	Pp03:22272039.22275403	462	TPS-b	DDMYD	DDLGTSKAE	—–	Cytoplasm
**PpTPS6**	Prupe.3G234300	5315	Pp03:22987731.22993046	830	TPS-e/f	DDFFD	NDIRSYQRE	—–	Cytoplasm
**PpTPS7**	Prupe.3G234400	4456	Pp03:23010884.23015340	694	TPS-e/f	—–	NEIRSYQKE	—–	Cytoplasm
**PpTPS8**	Prupe.3G234500	5251	Pp03:23019016.23024267	837	TPS-e/f	DDFFD	NEIRSYQKE	—–	Cytoplasm
**PpTPS9**	Prupe.4G125200	1799	Pp04:6849135.6850934	351	TPS-b	DDVYD	—————	—–	Cytoplasm
**PpTPS10**	Prupe.4G125300	8555	Pp04:6862449.6871004	554	TPS-b	DDVYD	NDLGTSTAE	—–	Cytoplasm
**PpTPS11**	Prupe.4G125500	2601	Pp04:6884416.6887017	544	TPS-b	—–	NDLGTSTAE	—–	Cytoplasm
**PpTPS12**	Prupe.4G126400	1186	Pp04:6943517.6944703	260	TPS-b	DDVYD	NDLATYQAE	—–	Cytoplasm
**PpTPS13**	Prupe.4G126600	2505	Pp04:6950949.6953454	557	TPS-b	DDVYD	NDLATYRAE	—–	Cytoplasm
**PpTPS14**	Prupe.4G128500	7267	Pp04:7067392.7074659	804	TPS-e/f	DDFFD	NDIQGFKRE	—–	Plasma membrane
**PpTPS15**	Prupe.4G128600	6515	Pp04:7077596.7084111	461	TPS-e/f	—–	—————	—–	Chloroplast
**PpTPS16**	Prupe.4G128700	2691	Pp04:7089282.7091973	275	TPS-e/f	—–	—————	—–	Cytoplasm
**PpTPS17**	Prupe.4G190700	3264	Pp04:11377002.11380266	550	TPS-a	NDIYD	NDMKSRQFE	—–	Cytoplasm
**PpTPS18**	Prupe.4G190800	1639	Pp04:11395603.11397242	358	TPS-a	DDIYD	—————	—–	Golgi apparatus
**PpTPS19**	Prupe.4G191000	3789	Pp04:11423113.11426902	563	TPS-a	NDIYD	NDIKSRQFE	—–	Cytoplasm
**PpTPS20**	Prupe.4G194100	3349	Pp04:11704060.11707409	561	TPS-a	DDIYD	DDVAGYKFD	—–	Chloroplast
**PpTPS21**	Prupe.4G194200	2988	Pp04:11749088.11752076	598	TPS-a	DDIYD	DDIAGSKFE	—–	Chloroplast
**PpTPS22**	Prupe.4G194300	2774	Pp04:11769339.11772113	557	TPS-a	DDIYD	DDIVSGQFE	—–	Chloroplast
**PpTPS23**	Prupe.4G194400	1957	Pp04:11801075.11803032	457	TPS-a	DDIYD	DDIISTNML	—–	Cytoplasm
**PpTPS24**	Prupe.4G194500	2484	Pp04:11819823.11822307	558	TPS-a	DDIYD	DDIVSTNFE	—–	Cytoplasm
**PpTPS25**	Prupe.4G194600	3426	Pp04:11828247.11831673	470	TPS-a	DDIYD	—————	—–	Mitochondria
**PpTPS26**	Prupe.4G197900	2843	Pp04:12117419.12120262	558	TPS-a	DDIYD	DDIVTSKFE	—–	Cytoplasm
**PpTPS27**	Prupe.4G198000	2268	Pp04:12134771.12137039	484	TPS-a	—–	DDLSGYEFE	—–	Cytoplasm
**PpTPS28**	Prupe.4G198200	2763	Pp04:12162746.12165509	582	TPS-a	DDIYD	DDIVSSKFE	—–	Cytoplasm
**PpTPS29**	Prupe.4G198500	2706	Pp04:12184503.12187209	521	TPS-a	DDIYD	—————	—–	Cytoplasm
**PpTPS30**	Prupe.4G198600	1917	Pp04:12190427.12192344	457	TPS-a	DDIYD	DDHSGYEFE	—–	Cytoplasm
**PpTPS31**	Prupe.4G198700	2072	Pp04:12194458.12196530	436	TPS-a	DDIYD	—————	—–	Chloroplast
**PpTPS32**	Prupe.4G199000	2255	Pp04:12240240.12242495	564	TPS-a	DDIYD	DDIVSSKFE	—–	Chloroplast
**PpTPS33**	Prupe.4G199200	4148	Pp04:12270980.12275128	562	TPS-a	DDIYD	DDMKSHKFE	—–	Cytoplasm
**PpTPS34**	Prupe.4G199300	1946	Pp04:12290148.12292094	457	TPS-a	DDIYH	DDIVSSKFE	—–	Cytoskeleton
**PpTPS35**	Prupe.4G199500	2497	Pp04:12346365.12348862	565	TPS-a	DDIYD	DDIVSSKFE	—–	Cytoplasm
**PpTPS36**	Prupe.4G200000	3696	Pp04:12416362.12420058	567	TPS-a	DDMYD	DDIVSSKFE	—–	Cytoplasm
**PpTPS37**	Prupe.4G200100	4574	Pp04:12426875.12431449	563	TPS-a	DDIYD	DDIVSSKFE	—–	Mitochondria
**PpTPS38**	Prupe.4G238400	9289	Pp04:15619531.15628820	524	TPS-e/f	NDFFD	—————	—–	Plasma membrane
**PpTPS39**	Prupe.4G238900	3386	Pp04:15686265.15689651	524	TPS-a	DDIYD	GDMKSHKFE	—–	Chloroplast
**PpTPS40**	Prupe.8G239900	6305	Pp08:21058645.21064950	697	TPS-c	—–	—————	DIDD	Cytoplasm

* Their subcelluar localization had been analyzed in previous studies (Liu et al., 2017; Wei et al., 2021).

# The subcellular localizations of PpTPSs were predicted with the online tool WOLFPSORT (https://wolfpsort.hgc.jp/).

**Figure 1:**
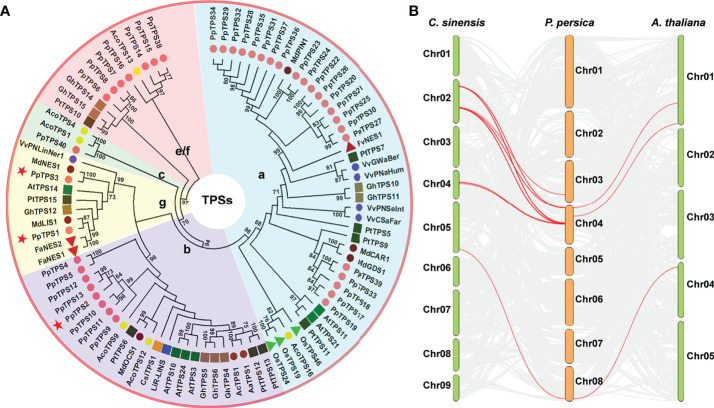
Evolutionary analysis of *PpTPS* genes. **(A)** A phylogenetic tree that was constructed using the full-length amino acid sequences of 40 TPSs from *Prunus persica* and 50 function-defined TPSs from *Arabidopsis thaliana*, kiwifruit (*Actinidia arguta*, *Actinidia chinensis*), pineapple (*Ananas comosus*), sweet orange (*Citrus sinensis*), strawberry (*Fragaria ananassa*, *Fragaria vesca*), cotton (*Gossypium hirsutum*), apple (*Malus domestica*), rice (*Oryza sativa*), poplar (*Populus trichocarpa*), and grapevine (*Vitis vinifera*). The phylogenetic tree was constructed with MEGA 11 using the Neighbor-joining method and the amino acid sequences were obtained from the previous studies (Aharoni et al., 2004; Tholl et al., 2005; Martin et al., 2010; Nieuwenhuizen et al., 2013; Nieuwenhuizen et al., 2015; Yoshitomi et al., 2016; Alquezar et al., 2017; Chen et al., 2017; Sun et al., 2017; Chen et al., 2018; Huang et al., 2018). The detailed accession numbers of all the TPS proteins were presented in **Supplementary Table 3**. Red stars denote three function-defined PpTPS proteins in previous studies. **(B)** The synteny analysis of *TPS* genes from *Prunus persica*, *Arabidopsis thaliana*, and *Citrus sinensis*.

### Gene structure and conserved protein domains of *PpTPSs*

**Figure 2:**
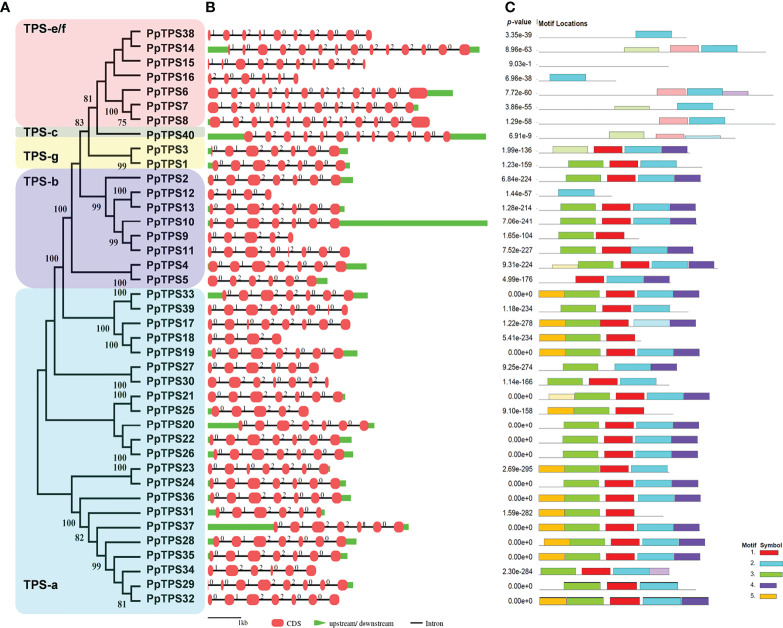
Structural analysis of *PpTPS* genes. **(A)** A phylogenetic tree of forty full-length PpTPS proteins. **(B)** A schematic graph shows the exon-intron organizations of *PpTPS* genes. **(C)** The organization of five protein motifs in PpTPSs predicted by MEME online tools. The detailed sequences of protein domains in PpTPSs were presented in **Supplementary Figure 1**.

To better understand the evolutionary relationships and structural characteristics of *PpTPSs*, the exon-intron organizations of these genes and the motifs of the deduced proteins were predicted using online tools. The *PpTPSs* that belong to class a, b, and g share similar gene structure with a smaller number of exons (4 to 8) and shorter coding DNA sequences (CDSs) (260 to 629 bp), whereas the members classified into class c and class e/f show a similar gene structure with a greater number of exons (6 to 14) and longer CDSs (275 to 837 bp) (**Table 1**, **Figures 2A, B**). The gene structure of *PpTPSs* directly determines the number and organization of protein domains/motifs, when the parameter is set as five conserved motifs to be predicted using the motif-based sequence analysis tools in the MEME Suite (http://meme-suite.org/meme/) (**Supplementary Figure 1**). Thirty PpTPSs contain the conserved DDxxD motif (Motif 2 in **Figure 2C**), four PpTPSs have mutant DDxxD motif, and six PpTPSs have thoroughly lost this motif (**Table 1**, **Figure 2C**). Even among 30 PpTPSs with DDxxD motif, 5 PpTPSs have lost the NSE/DTE motif, which means that only 25 PpTPSs might have kaurene synthase activities, because both the DDxxD and NSE/DTE motifs are essential to the metal dependent ionization of the prenyl diphosphate substrate. In addition, the unique class c TPS, PpTPS40, has a DxDD motif that is important for copalyl synthase activities, which suggests that it might play a role in the protonation-initiated cyclization of geranylgeranyl diphosphate to CPP (**Table 1**).

### The expression profiles of *PpTPSs* in the shoot of ‘Huyou018’ inoculated by *B. dothidea*

**Figure 3:**
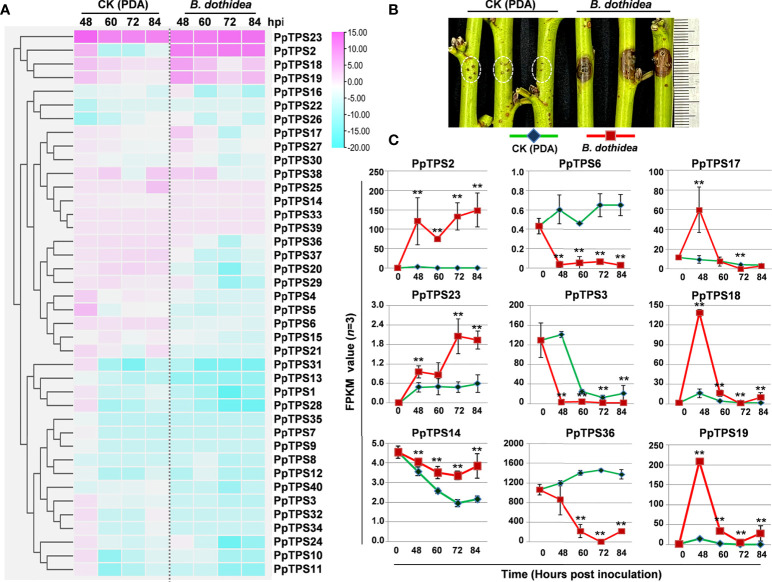
Expression profiles of *PpTPS* genes in peach shoots post *B. dothidea* inoculations determined by RNA sequencing. **(A)** A heatmap shows the relative transcript levels (FPKM value) of forty *PpTPS* genes in the shoots of ‘Huyou018’ after PDA medium (Mock) and *B. dothidea* inoculations. The color scale represents log(fold change, 2) values of *PpTPSs* transcript levels at each time point (48 h, 60 h, 72 h, and 84) compared with 0 h after each treatment, with pink denoting increased transcript abundance and blue denoting decreased transcript abundance. **(B)** The representative photograph shows the phenotype of peach shoots at 48 h after *B. dothidea* inoculations. **(C)** Line graphs show the detailed FPKM value of nine selected *PpTPS* genes after PDA medium (Mock) and *B*. *dothidea* inoculations. The asterisks denote that the FPKM values of a gene in *B*. *dothidea* inoculations were significantly different with that in Mock (*p*<0.05). The detailed FPKM values of *PpTPSs* in pathogen inoculation assays were presented in **Supplementary Table 4**.

To explore the potential roles of *PpTPSs* in the defense response of peach against fungus that induces peach gummosis, we analyzed the transcript levels (FPKM values) of 40 *PpTPSs* in the shoot of ‘Huyou018’ after inoculation with *B. dothidea via* RNA sequencing (**Supplementary Table 4**). The majority of *PpTPSs* showed down-regulation at the transcription level, and some *PpTPSs* showing elevated transcription levels in mock treatment (potato dextrose agar, PDA, medium inoculations) were down-regulated at the transcription level after inoculation with pathogens (**Figure 3A**). Several *PpTPSs* including *PpTPS2*, *PpTPS17*, *PpTPS18*, *PpTPS19*, and *PpTPS23* were up-regulated at the transcription level post inoculation, but they showed differences (**Figure 3A**). For example, *PpTPS2* and *PpTPS23* were gradually up-regulated at the transcription level, whereas *PpTPS17*, *PpTPS18*, and *PpTPS19* were up-regulated at 48 h post inoculation (hpi), and then they reduced their expression until 84 hpi (**Figures 3B, C**).

**Figure 4:**
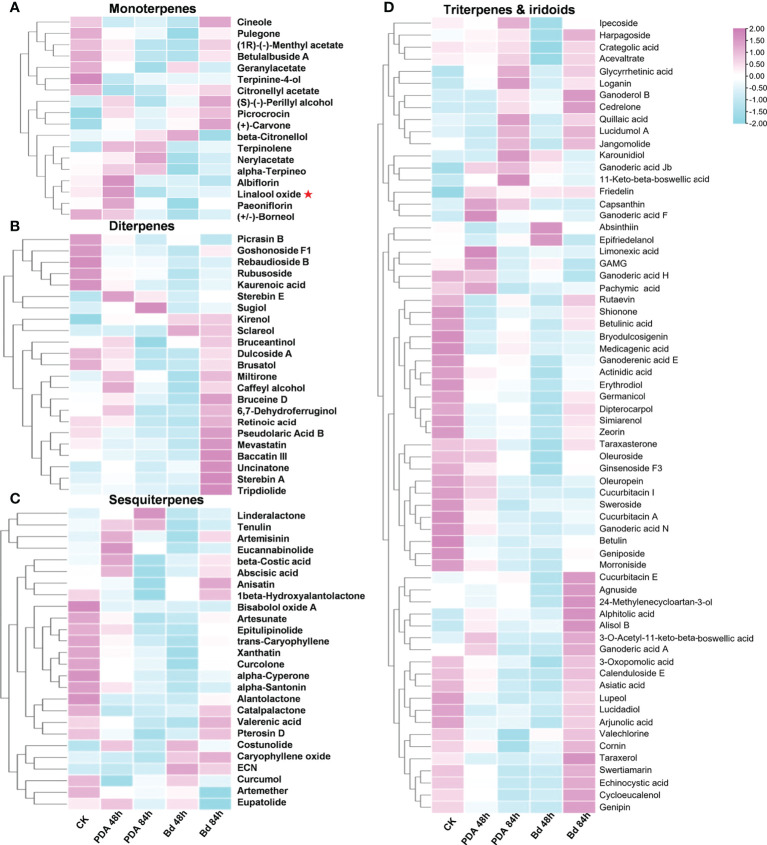
The relative contents of 133 terpenoids in peach shoots post *B. dothidea* inoculations determined by UHPLC-MS. **(A–D)** Heatmaps show the relative content of eighteen monoterpenes **(A)**, twenty-three diterpenes **(B)**, twenty-six sesquiterpenes **(C)**, and 65 triterpenes and iridoids **(D)** in the shoot of ‘Huyou018’ after PDA medium (Mock) and *B. dothidea* inoculations, respectively. The color scale represents relative contents of terpenoids at each time point (0 h, 48 h, 84 h) with pink denoting relatively higher terpenoids contents and blue denoting relatively lower terpenoids contents. A red asterisk in **(A)** points out the linalool oxide. The detailed relative contents of 133 terpenoids were presented in **Supplementary Table 5**.

To further verify the relationship between the transcriptions of *PpTPSs* with the accumulation of terpenoid metabolites in the shoot post inoculation with *B. dothidea*, we measured the content of multiple metabolites in the same batches of samples used for RNA sequencing. A total of 133 terpenoid metabolites were detected, including 18 monoterpenes, 23 diterpenes, 26 sesquiterpenes, 51 triterpenes, 14 iridoids, and one capsanthin (**Figure 4**, **Supplementary Table 5**). Based on the peak areas of each metabolite obtained from UHPLC-MS assays, we found that the relative contents of many detected terpenes (22% of monoterpenes, 52% of diterpenes, 19% of sesquiterpenes, 33% of triterpenes, and 21% of iridoids) were elevated in response to the inoculation with *B. dothidea*, especially at 84 hpi. The relative contents of some terpenoids also changed after mock treatments.

### Transcript accumulation patterns of *PpTPSs* at different fruit developmental stages

**Figure 5:**
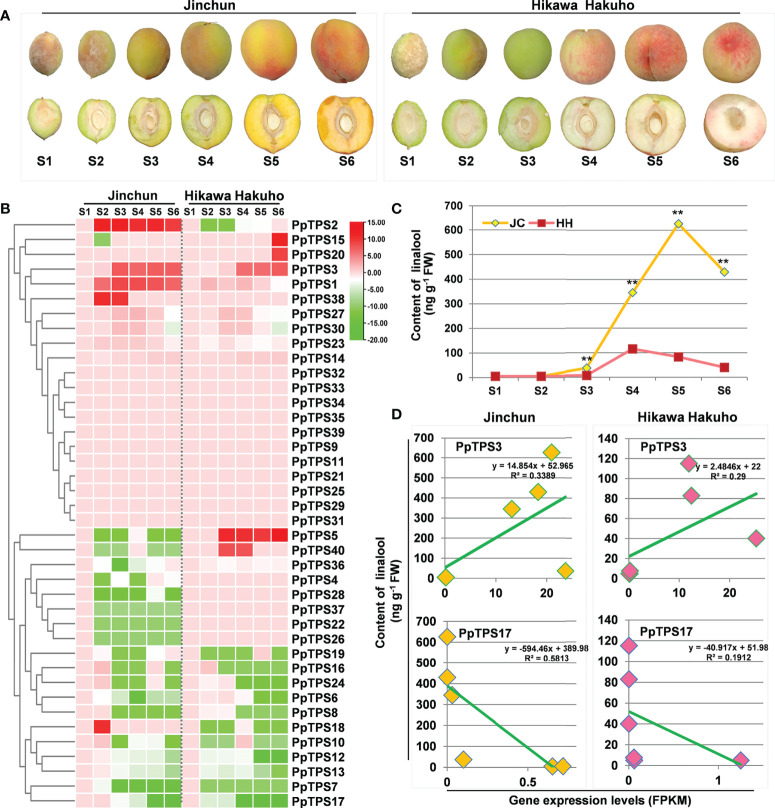
Expression profiles of *PpTPS* genes during fruit development determined by RNA sequencing. **(A)** Representative photographs show the peach fruits at each sampling time point. **(B)** A heatmap shows the relative transcript levels (FPKM value) of forty *PpTPSs* in fruit pulps of ‘Jinchun’ (JC) and ‘Hikawa Hakuho’ (HH) at different stages of fruit development and ripening stages (35 d (S1), 50 d (S2), 65 d (S3), 80 d (S4), 90 d (S5), 95 d (S6)). The color scale represents log (fold change, 2) values of *PpTPSs* transcript levels at each development stages compared with S1, with red denoting increased transcript abundance and green denoting decreased transcript abundance. The detailed FPKM values of *PpTPSs* during fruit development were presented in **Supplementary Table 6**. **(C)** A line graph shows the content of linalool in fruit pulps of ‘Jinchun’ and ‘Hikawa Hakuho’ at different stages of fruit development. The asterisks denote that the linalool contents in ‘Jinchun’ were significantly higher than that in ‘Hikawa Hakuho’ at each sampling time site (*p*<0.05). **(D)** Line graphs show the linear correlations between the relative transcript levels of *PpTPSs* (*PpTPS3* and *PpTPS17*) and the contents of linalool in fruit pulps of ‘Jinchun’ and ‘Hikawa Hakuho’ during fruit development.

To explore the potential function of *PpTPSs* in the development of peach fruits, we analyzed the transcript levels (FPKM values) of 40 *PpTPSs* during fruit development based on RNA sequencing data (**Supplementary Table 6**, **Figure 5A**). Approximately half of the *PpTPSs* showed down-regulated transcript levels at different sampling times compared to the first sampling time (S1); thus, these *PpTPS* genes could reduce their expression during peach fruit development (**Figure 5B**). Fifteen *PpTPSs* showed consistent transcript levels during the entire process of peach fruit development, suggesting that they are not directly associated with fruit development (**Figure 5B**). However, five *PpTPSs* exhibited elevated transcript levels at the middle or late stages of peach fruit development either in yellow-fleshed cultivar ‘Jinchun’ or in white-fleshed cultivar ‘Hikawa Hakuho’; thus, these *PpTPSs* can contribute to the biosynthesis of aroma-related terpenoids (**Figure 5B**). Most *PpTPSs* showed similar expression profiles in these two peach cultivars, whereas several *PpTPSs* exhibited distinct expression characteristics. For example, the transcript levels of *PpTPS2* and *PpTPS18* were up-regulated in ‘Jinchun’ but reduced at the same sampling time points in ‘Hikawa Hakuho’ (**Figure 5B**). In contrast, the transcript levels of *PpTPS5* and *PpTPS40* were up-regulated in ‘Hikawa Hakuho’ but down-regulated in ‘Jinchun’. The transcript levels of *PpTPS1* and *PpTPS38* only showed up-regulation in ‘Jinchun’, but those of *PpTPS15* and *PpTPS20* only showed up-regulation in ‘Hikawa Hakuho’ (**Figure 5B**). These results suggest that the expression of *PpTPSs* is cultivar-specific.

To further verify the relationship between the transcriptions of *PpTPSs* and the accumulation of terpenoid metabolites in peach fruits, we measured the content of linalool, the predominant terpene accumulated in peach fruits, in the same batches of samples used for RNA sequencing. The linalool content gradually elevated during the early stages of fruit development both in ‘Jinchun’ and ‘Hikawa Hakuho’, and peaked at S5 and/or S4, respectively, then decreased during fruit ripening stages (**Figure 5C**). Obviously, the fruit pulp of ‘Jinchun’ contains higher linalool content than that of ‘Hikawa Hakuho’ at all sampling times (**Figure 5C**). Correlation analysis demonstrated that the expression profiles of *PpTPS3* exhibited positive linear correlations with the linalool content both in ‘Jinchun’ and ‘Hikawa Hakuho’ (**Figure 5D**). However, the expression profiles of *PpTPS17* exhibited negative linear correlations with the linalool content in both cultivars (**Figure 5D**).

### The subcellular locations of three PpTPSs in the leaves of *Nicotiana benthamiana*

**Figure 6:**
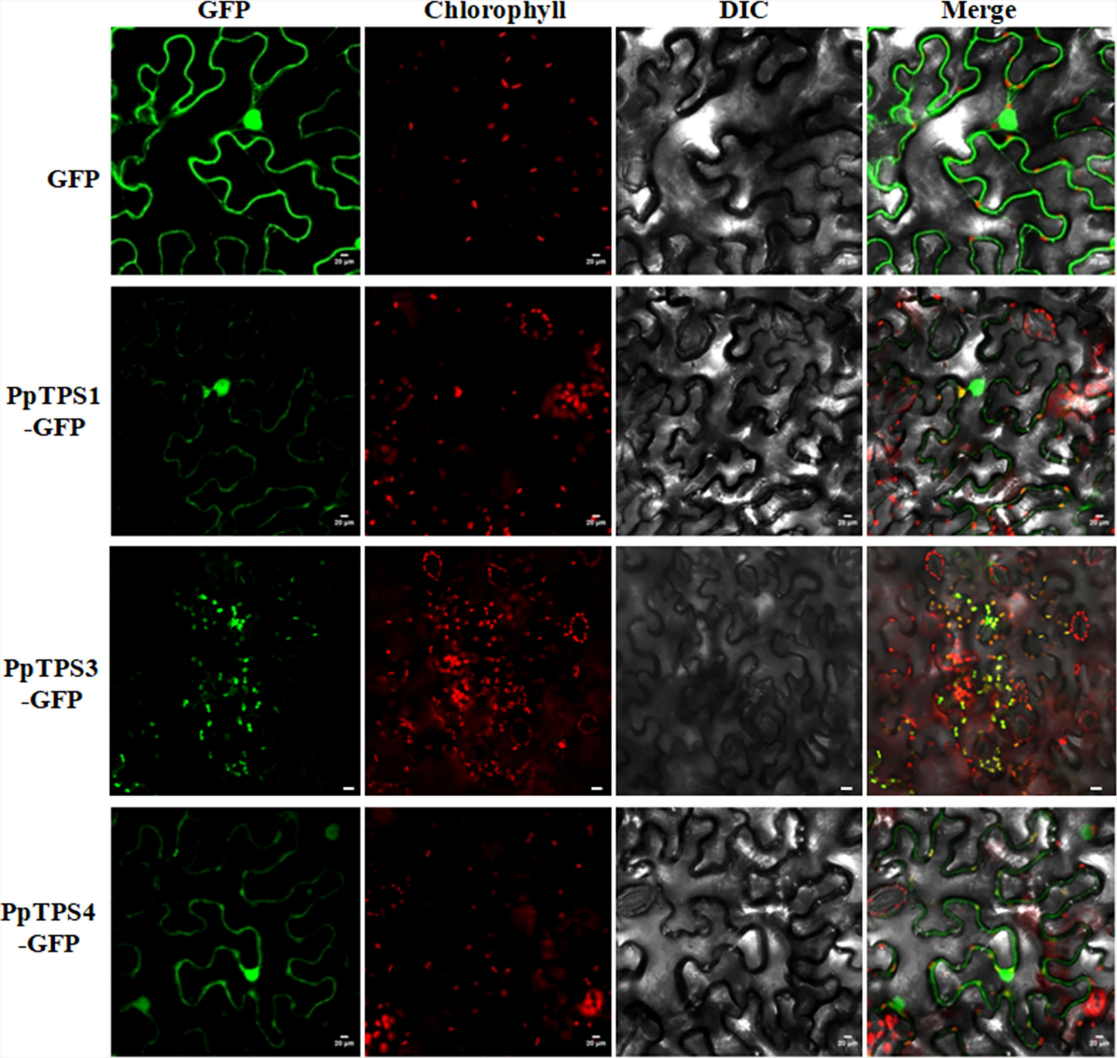
Subcellular localizations of three PpTPS-GFP fusion proteins in the leaves of *Nicotiana benthamiana*. The primers used for gene cloning were listed in **Supplementary Table 1**.

For TPS proteins, the subcellular localization determine their substrates availabilities, therefore, it should be an essential character of each TPS individual. To preliminarily investigate the subcellular localization of PpTPSs, we selected three representative *PpTPSs*, including *PpTPS1*, *PpTPS3*, and *PpTPS4*, that showed elevated transcription levels post pathogen inoculations or during the fruit development process, fused their CDS with *GFP* gene, and then transiently expressed these fusion genes in the leaves of tobacco. As shown in **Figure 6**, PpTPS3-GFP showed specific chloroplast localizations in the leaves of tobacco, while PpTPS1-GFP and PpTPS4-GFP not only exhibited co-localization with chloroplasts, but also partially distributed in nucleus and cytoplasm.

### Antifungal activities of linalool in PDA medium

**Figure 7:**
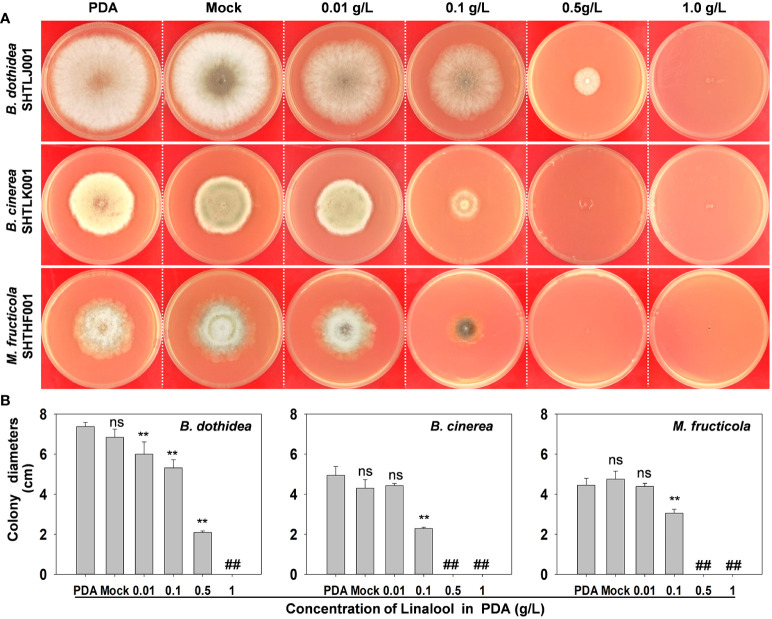
Antifungal activities of linalool in PDA medium. **(A)** Representative photographs show the growth of three pathogens on the PDA mediums containing different concentrations of linalool at 3 days post inoculation.**(B)** Statistic of the colony diameters of three pathogens on PDA mediums at 3 days post inoculation. The asterisks denote that the colony diameters of each pathogen in those PDA mediums containing linalool were significantly lower than that in PDA mediums without ethanol (Mock) and linalool (*p*<0.05), ‘##’ indicates no data were obtained and ‘ns’ means the difference was not significant.

To examine whether the metabolites of *PpTPSs* coding enzymes are involved in peach defense responses against fungi or not, we analyzed the antifungal activities of linalool in PDA medium. As shown in **Figure 7A**, though lower levels of linalool did not suppress the hyphal growth of fruit parasitic pathogens (*B. cinerea* and *M. fruticola*), but the elevated linalool contents (>0.1 g/L) could significantly limit the hyphal growth of all the three main peach pathogens in PDA medium (**Figure 7B**). This result suggested that linalool might have broad-spectrum antifungal activities.

### Identification of *cis*-regulatory elements in the promoter of *PpTPSs*

**Figure 8:**
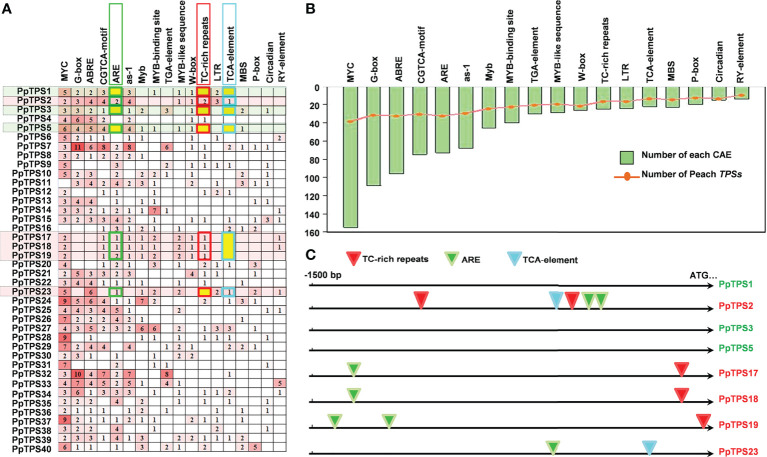
Distribution of *cis*-acting regulatory elements in the promoter of *PpTPS* genes. **(A)** A heatmap shows the number of different *cis*-acting regulatory elements in the promoter of forty *PpTPSs.* **(B)** A bar graph shows the total number of each *cis*-acting regulatory element identified in the promoter of forty *PpTPSs*. The curve point denotes the number of *PpTPSs* having each *cis*-acting regulatory element. **(C)** A schematic shows the distribution of three SA-associated *cis*-acting regulatory elements in the promoters of eight selected *PpTPSs*.

To explain the likely mechanisms of the distinct expression patterns of different *PpTPS* members either in defense response or in fruit floral-note aroma formation, we analyzed the distributions of *cis*-acting regulatory elements in the promoter regions of 40 *PpTPS* genes. Over 15 *cis*-acting regulatory elements were identified, and they were widely distributed in these promoters. The top six identified elements are MYC (-CATTTG-), G-box (-CACGTT/C-), ABRE (-ACGTG-), CGTCA-box (-CGTCA-/-TGACG-), ARE (-AAACCA-), and as-1 (-TGACG-) (**Figures 8A, B**). All of them were distributed in the promoters of at least 30 *PpTPSs*. In addition, multiple types of MYB transcription factor binding sites, including ARE, Myb (-TAACTG-), MYB-binding site (-CAACAG-), MYB-like sequence (-TAACCA-), and MBS (-CAACTG-) were identified (**Figure 8B**). The comparison of the promoter sequences of *PpTPS1*, *PpTPS2*, *PpTPS3*, *PpTPS17*, *PpTPS18*, and *PpTPS19* showed that the presence or absence of ARE, TC-rich repeats (-GTTTTCTTAC-), and TCA-element (-TCAGAAGAGG-) might impact the expression profiles of *PpTPSs* in the shoot after inoculation with *B. dothidea* (**Figure 8C**).

## Discussion

Plant VOCs can affect the disease resistance and economical value of agricultural plants, especially horticultural crops (Aharoni et al., 2004; Lin et al., 2017; Liu et al., 2017; Chen et al., 2018; Huang et al., 2018; Wei et al., 2021). Owing to the unique function of TPSs in the biosynthesis of terpenoids, which is the largest group of plant-released VOCs, studies similar to the present analysis in *Arabidopsis*, tomato (*Solanum lycopersicum*), carrot (*Daucus carota*), cotton (*Gossypium hirsutum*), pineapple (*Ananas comosus*), apple (*Malus domestica*), grapevine (*Vitis vinifera*), tea (*Camellia sinensis*), holy basil (*Ocimum sanctum*), and *Cannabis sativa* have identified numerous *TPS* genes (Aubourg et al., 2002; Martin et al., 2010; Falara et al., 2011; Nieuwenhuizen et al., 2013; Alquezar et al., 2017; Chen et al., 2017; Keilwagen et al., 2017; Huang et al., 2018; Kumar et al., 2018; Allen et al., 2019; Yu et al., 2020; Zhou et al., 2020; Zhang et al., 2021). However, except for the preliminary identification and evolution analysis of *TPS* genes in Rosaceae and few studies reporting the roles of three *PpTPSs* (*PpTPS1*, *PpTPS2* and *PpTPS3*) in the biosynthesis of flavor-related linalool and (*E*,*E*)-*α*-farnesene, respectively, limited information is available about this important gene family in peach (Liu et al., 2017; Wei et al., 2021; Zhang et al., 2021; Wei et al., 2022; Zhang et al., 2022). In this study, we investigated expression profiles of peach *TPS* genes in current-year shoots post *B. dothidea* inoculations and/or in fruit pulps during fruit development, and tried to explore the potential roles of PpTPSs and/or their metabolites in disease resistance.

*TPS* genes widely found in bacteria, fungi, and plants are likely derived from a common ancestor gene and then undergo species-specific expansion (Chen et al., 2011). Compared to other higher plants, peach has a smaller quantity of *TPS* genes (Martin et al., 2010; Nieuwenhuizen et al., 2013; Zhou et al., 2020; Zhang et al., 2021) (**Table 1**, **Figure 1A**) because Prunoideae has not experienced recent whole-genome duplication events (Zhang et al., 2021). In addition, the *TPS* genes intensively distribute in several gene clusters of three chromosomes in peach genome (**Table 1**, **Figure 1A**), indicating that these chromosome regions harboring *PpTPS* genes have not undergone large-scale segment duplications or recombination in the form of crossing over between nonhomologous chromosomes. Alternatively, tandem duplications may be the cause of *TPS* gene family expansion (copy number variations) in peach. We showed that the gene structures of *PpTPSs* were highly similar but the loss of exons in some duplicated *PpTPSs* led to variations in key protein motifs or missing protein motifs (**Figures 2A, B**). Being similar to other angiosperm plants, though 25 deduced PpTPSs contain the intact KS activity center, only the unique class c member PpTPS40 still harbors a CPS activity center (**Table 1**), which means that the bifunctional diterpene synthase could be absent in peach.

The distinctive and rich flavor of peach fruit, which is a key trait of peach attracting consumers, is attributed to the mixture of volatiles, sugars, and acids. To date, more than 100 volatile chemicals have been identified in peach fruit, and linalool is the main odorant that affects fruit aroma and consumer preference (Eduardo et al., 2010). In this study, we observed that linalool is a dominant terpenoid detected in fruit pulps of two peach cultivars (‘Jinchun’ and ‘Hikawa Hakuho’) using GC-MS (**Figure 5C**). Among 40 *PpTPSs*, the transcript level (FPKM) of *PpTPS3* showed positive linear correlations with the linalool content, suggesting that *PpTPS3* can directly contribute to the biosynthesis of linalool (**Figure 5D**). In a recent study, this gene has been identified to encode a linalool synthase in peach fruit (Wei et al., 2021). *PpTPS2* showed distinct expression profiles in ‘Jinchun’ and ‘Hikawa Hakuho’ during fruit development and has been identified to encode an UV-B-induced sesquiterpene synthase that promotes the accumulation of (*E,E*)-*α*-farnesene in peach fruits (Liu et al., 2017) (**Figure 5D**).

Fungi-induced perennial gummosis on trunks and branches, caused by multiple fungi classified as *Botryosphaericeae* (*Lasiodiplodia theobromae*, *Botryosphaeria dothidea*, *Diplodia seriata*, *Neofusicoccum parvum*), is a major problem in peach orchards worldwide (Wang et al., 2011). Several studies reported that the overexpression of *TPS* genes could improve the resistance of plants against bacteria, fungi, and nematodes, owing to the ectopic accumulations of different terpenoids, including linalool, (*S*)-limonene, and gamma-terpinene (Shimada et al., 2014; Yoshitomi et al., 2016; Lin et al., 2017; Shimada et al., 2017; Chen et al., 2018; Huang et al., 2018). In this study, most of the 40 *PpTPS* genes showed changed transcript levels and over 100 terpene-related metabolites exhibited altered contents in the shoots post *B. dothidea* inoculation, indicating the terpenoid-related transcription and metabolism were strongly induced during fungal gummosis (**Figures 3** and **4**). Although over 30 *PpTPS* genes were down-regulated at the transcription levels after inoculation with pathogens, several *PpTPS* genes (*PpTPS2*, *PpTPS17*, *PpTPS18*, *PpTPS19*, and *PpTPS23)* were gradually up-regulated and/or were predominantly up-regulated at the early stages (48 hpi) of pathogen infections, suggesting their potential importance in this bioprocess (**Figure 3C**). While most of the detected metabolites showed reduced relative contents at 48 hpi compared to 0 hpi, over half of these diterpenes and triterpenes displayed substantial accumulations at 84 hpi (**Figure 4**), suggesting that the metabolism of terpenoids were finely regulated in peach shoots during pathogen infections.

To support the above speculations, we also examined the antifungal activities of linalool in PDA medium, and found that high concentrations (0.1 g/L to 1.0 g/L) of linalool could fully limit the hyphal growth of *B. dothidea*, *B. cinerea*, and *M. fruticola*, which cause devastating diseases in peach (**Figures 7A, B**). This result suggests that linalool and/or it-related terpenes could be effective chemical weapons of peach against necrotrophic pathogens. However, the transcription of *PpTPS3*, the most important contributor of linalool biosynthesis, was down-regulated during *B. dothidea* inoculations compared to PDA treatments in the shoot of ‘Huyou018’ (**Figure 3C**), indicating the defense-related metabolisms of peach could be hijacked by pathogens *via* transcriptional regulations.

TPSs are vital terminal enzymes catalyzing the formation of plant terpenoids by using various prenyl diphosphate precursors as substrates, but the transcription of *TPS* genes can be controlled by many transcription factors. For example, in flowers of *Arabidopsis*, AtMYC2 interacts with DELLA proteins to regulate sesquiterpene synthase gene expression (Hong et al., 2012). In flowers of monocotyledonous plant *Freesia hybrida*, two interacted transcription factors, FhMYB21L2 and FhMYC2, aggressively regulate the transcription of linalool synthesis gene *FhTPS1* (Yang et al., 2020). In the leaves of sweet orange, CiMYB42 regulates the expression of *CiOSC* by binding to the -TTGTTG- sequence (type II MYB core) (Zhang et al., 2020). In peach fruit, transcription factor PpbHLH1 and PpERF61 activate *PpTPS1* and *PpTPS3* expression by directly binding to E-box and DRE/CRT *cis*-element in their promoters, respectively (Wei et al., 2021; Wei et al., 2022). These results suggest that bHLH, ERF and MYB transcription factors can be the major upstream regulators of *TPS* genes. In this study, we also observed that MYC2, G-box, ABRE, CGTCA-motif, and multiple types of MYB-binding sites are widely distributed in the promoters of almost all the *PpTPSs* (**Figures 8A, B**). ABRE is not directly associated with bHLH and/or MYB transcription factors, but ABA signaling is highly associated with these transcription factors, which indirectly affects the biosynthesis of terpenoids *via* ABRE (Abe et al., 2003). In addition, SA-related *cis*-acting regulatory elements, including as-1, ARE, TCA-element, and TC-rich repeats, might also affect the distinct expression profiles of *PpTPSs*. For example, the presence or absence of ARE, TC-rich repeats, and TCA-elements is associated with the pathogen-induced up-regulations after inoculation with *B. dothidea* (**Figure 8C**). Although the experimental evidences uncovering the relationships between phytohormone signalings and terpenes biosynthesis is lacking, we speculated that the cross-talks between different phytohormone signaling pathways could fine tune the terpenes biosynthesis *via* various transcription factors. Therefore, more efforts are required to elucidate the roles of divergent phytohormone and transcription factor in regulating terpene biosynthesis in the further studies.

## Conclusion

In this study, we identified, classified, and named *TPS* family genes in *P. persica* and assessed the expression profiles of them in current-year shoots post *B. dothidea* inoculations and/or in fruit pulps during fruit development *via* RNA sequencing. Being highly similar with previous studies, *PpTPS1*, *PpTPS2*, and *PpTPS3* exhibited dominant transcript levels and significantly elevated their transcript levels during fruit development and ripening in two peach cultivars, suggesting their essential roles in the formation of terpene-derived peach flavors and fruit quality. Moreover, we also observed that over 20 *PpTPSs* showed reduced transcript levels in defense responses against *B. dothidea*, and 10 *PpTPSs* exhibited elevated transcript levels during the same process. Meanwhile, the results of UHPLC-MS and GC-MS analyses revealed that the contents of different terpenoids were altered in defense responses of peach. These results suggest the potential importance of *PpTPSs* in peach defense responses. Notably, the transcription levels of *PpTPS3* exhibited positive linear correlations with the linalool contents in fruit pulps, suggesting its important roles in linalool biosynthesis. In addition, we also provide evidences to support the effective antifungal activities of linalool in PDA medium. Taken together, our results provide a foundation for further studies to explore the function of *PpTPS* genes in response to biotic stress stimuli; however, additional studies are required to elucidate the detailed function of each TPS protein in this economically important fruit crop.

## Data availability statement

The datasets presented in this study can be found in online repositories. The names of the repository/repositories and accession number(s) can be found below: BioProject accession number: PRJNA828349.

## Author contributions

XL and ZY initiated the project, designed the experiment. MS, JD, HZ and XZ collected the samples. YH, XL and MZ analyzed and processed the raw data. YH and XL drafted the manuscript. XL and ZY reviewed the manuscript. All authors contributed to the article and approved the submitted version.

## Funding

This work was supported by funds from Shanghai Science and Technology committee Rising-Star Program (19QB1404600), the National Key Research and Development Program of China (2019YFD1000801), the Outstanding Team Program of Shanghai Academy of Agricultural Science (Grant No. 2022–004).

## Acknowledgments

We are grateful for the help of Shanghai BIOTREE Biological Technology Co., Ltd. (Shanghai, China) in linalool and terpenoids measurement.

## Conflict of interest

The authors declare that the research was conducted in the absence of any commercial or financial relationships that could be construed as a potential conflict of interest.

## Publisher’s note

All claims expressed in this article are solely those of the authors and do not necessarily represent those of their affiliated organizations, or those of the publisher, the editors and the reviewers. Any product that may be evaluated in this article, or claim that may be made by its manufacturer, is not guaranteed or endorsed by the publisher.
