# Supplementary figures and images for: Genome-wide analysis of terpene synthase gene family to explore candidate genes related to disease resistance in Prunus persica
Source: Front Plant Sci. 2022 Oct 31;13:1032838. doi: 10.3389/fpls.2022.1032838 (PMC9660250; doi:10.3389/fpls.2022.1032838)

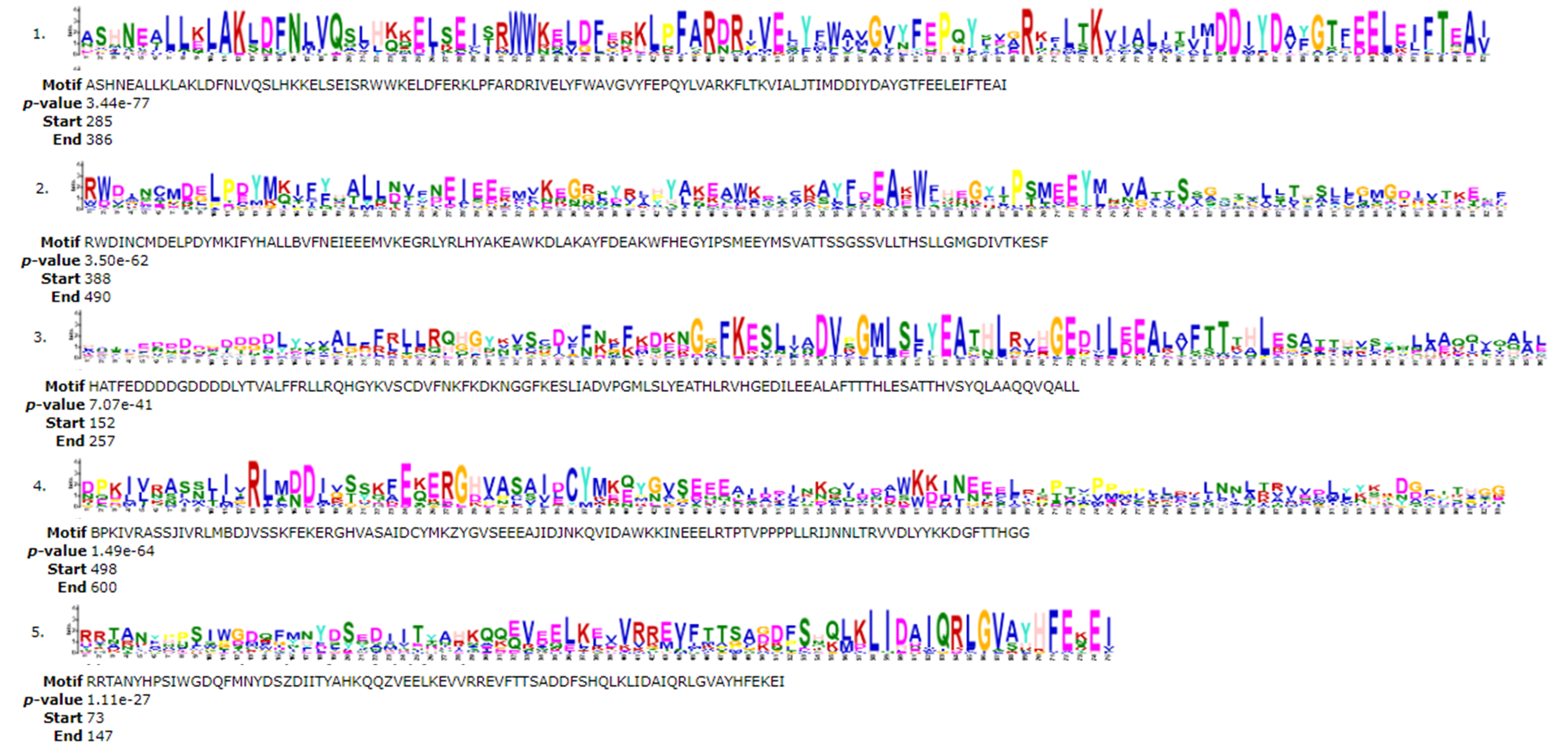

Supplement: Supplementary file 1 [file Image_1.png]
